# Clinical outcome of OviTex reinforced tissue matrix in hernia repair: A systematic review and meta-analysis

**DOI:** 10.5339/qmj.2025.22

**Published:** 2025-02-17

**Authors:** Ahmad R. Al-Qudimat, Seif B. Altahtamouni, Kalpana Singh, Omar M. Aboumarzouk, Mohamed Elakkad

**Affiliations:** ^1^Surgical Research Section, Department of Surgery, Hamad Medical Corporation, Doha, Qatar; ^2^Department of Public Health, College of Health Sciences, QU Health, Qatar University, Doha, Qatar; ^3^ Department of Nursing, Hamad Medical Corporation, Doha, Qatar; ^4^College of Medicine, QU Health, Qatar University, Doha, Qatar; ^5^School of Medicine, Dentistry and Nursing, University of Glasgow, Glasgow, UK; ^6^Department of Surgery, Hamad Medical Corporation, Doha, Qatar *Correspondence: Ahmad R. Al-Qudimat. Email: aalqudimat@hamad.qa

**Keywords:** Hernia, OviTex, repair, clinical outcomes

## Abstract

**Background:** This review provides a comprehensive and current overview of the clinical outcomes associated with the use of OviTex reinforced tissue matrix (RTM) in hernia repair. **Methods:** We conducted a systematic review and meta-analysis according to the Preferred Reporting Items for Systematic Reviews and Meta-Analyses (PRISMA) guidelines. Our search included research publications related to clinical outcomes involving the use of OviTex RTM in hernia repair up to August 2023. We extensively examined and extracted relevant data from databases such as Embase, PubMed, and Scopus. The meta-analysis included comparisons related to body mass index (BMI) in hernia treatment, primary abdominal wall hernias treated with OviTex, and other relevant factors. The quality of the included studies was assessed using the MINORS (Methodological Index for Non-Randomized Studies) scale. Our systematic review protocol was registered with PROSPERO (International Prospective Register of Systematic Reviews) under registration number CRD42023456009. **Results:** A total of 9 observational studies involving 990 patients from three countries (USA, Netherlands, and Germany) were included in our study. Pooled results show that the risk of the OviTex group was lower than that of the comparison group (pooled risk ratio (RR) = 0.84; 95% confidence interval (CI): 0.67–1.05; *Z* = -1.514; *p* = 0.13). The prevalence rate of primary abdominal wall hernia among the included studies ranged from a minimum of 43% (95% CI: 30–58) to a maximum of 81% (95% CI: 64–91%), the risk was not significantly higher in the comparison group compared with the OviTex group (pooled RR = 1.11; 95% CI: 0.29–4.30; *Z* = 0.155; *p* = 0.877), the prevalence of laparoscopic surgery was 12% (95% CI: 6–19%), the BMI was favorable in the comparison group but was not statistically significant (mean difference = 25; 95% CI: -0.02, 0.52; *p* = 0.073). **Conclusion:** OviTex RTM has shown promising outcomes in abdominal wall reconstruction and hernia repair. However, it is crucial to conduct further research and clinical studies to confirm these findings and unlock the capabilities of OviTex across different medical scenarios.

## Introduction

Abdominal wall hernias can be defined as a protrusion or prolapse through the abdominal wall.^1^ They are a common clinical condition that requires surgical intervention and repair to prevent a wide range of complications such as irreducibility, obstruction, and strangulation. Inguinal hernias are the most frequently occurring abdominal wall hernias, constituting 70–75% of cases, followed by femoral and umbilical hernias at 6–17% and 3–8.5%, respectively.^2^ Over 20 million hernia repair procedures are performed worldwide yearly, making it the most common surgical intervention globally.^3^ Abdominal wall hernias were traditionally closed with primary suture repair. However, in 1958, Dr. Usher introduced the use of polypropylene mesh, which led to the development of the Lichtenstein repair 30 years later and popularized mesh for hernia repair.^4^ This new technique, using polymer-based mesh, reduced the rates of recurrence and infection.^5^ A multicenter randomized clinical trial by Rosen et al. compared biological and synthetic meshes in single-stage repair of contaminated ventral hernias. The study showed a significant reduction in the risk of hernia recurrence in the synthetic mesh group (hazard ratio 0.31; 95% CI: 0.23–0.42; *p* < 0.001).^6^ However, the use of synthetic mesh comes with its complications, such as surgical site infections (SSIs), seroma formation, foreign body reactions, bowel adhesions, and fistulas.^7–9^

While synthetic polymer-based meshes have been widely adopted due to their durability and reduced recurrence rates, they also have significant drawbacks, such as increased risk of infections, seroma formation, bowel adhesions, foreign body reactions, and fistulas.^7–9^ These complications often arise due to the strong inflammatory response and the lack of biological integration of the synthetic material with the host tissue.^2^ On the contrary, biological meshes, derived from mammalian tissues, provide a more biocompatible scaffold that promotes repopulation and revascularization of host tissue, thereby reducing the inflammatory response.^2^ However, these biological options are often more expensive and may not provide the same long-term mechanical strength required for larger or more complex hernia repairs.^2^

Recently, a new type of mesh called reinforced tissue matrix (RTM) has emerged, which has the potential to increase abdominal wall support and reduce the inflammatory response, which can reduce the recurrence and infection rates.^10^ OviTex® (TELA Bio Inc., Malvern, PA, USA) is a type of sterile permanent RTM composed of extracellular matrix (ECM) derived from biological tissue (ovine) and minimal polymer reinforcement.^10^ A study by Overbeck et al. evaluated the use of RTM in full-thickness midline abdominal wall defects in non-human primates compared with other clinically used biological and synthetic meshes.^11^ The results showed better outcomes for RTM in terms of inflammatory response, cellular infiltration, remodeling time, and the formation of new morphologically functional collagen.^11^ Although data on the use of OviTex in human abdominal wall repair are limited, low hernia recurrence rates were observed with no mesh-related complications.^12^

OviTex® represents a significant advancement in mesh technology by combining the benefits of both synthetic and biological materials. As a hybrid mesh, OviTex incorporates an ovine ECM with minimal polymer reinforcement, providing a balance between mechanical durability and biological integration.^1,2^ The biological component supports better cellular infiltration and tissue remodeling, while the synthetic fibers ensure that the mesh retains its structural integrity over time, even in complex or contaminated areas. This hybrid design is intended to overcome the limitations of fully synthetic meshes, particularly the issues of foreign body reactions and long-term complications.^2^

This requires further research to validate the use of RTM in hernia repair. A prospective, single-arm, multi-center clinical trial based on the 24-month results of the BRAVO study evaluated the clinical outcomes of ventral hernia patients treated with OviTex® 1S permanent RTM.^10^ The study showed that the use of OviTex is a viable option in the management of ventral hernias. The use of OviTex RTM appears to show promising results in reducing the risk of recurrence and complication rates. However, more research with larger sample sizes, longer follow-up periods, and higher statistical power is needed to reach consensus on the use of RTM and minimize the gap in the current literature. To this end, we conducted a comprehensive systematic review and meta-analysis that included all current and available data on this topic to examine the safety and efficacy of RTM use in abdominal wall repair.

## Methods

This meta-analysis was conducted in accordance with the Preferred Reporting Items for Systematic Reviews and Meta-Analyses (PRISMA) guidelines.^13^

### Search strategy

A comprehensive search was performed in the Embase, PubMed, and Scopus databases published up to August 1, 2023 using the terms hernia patients, RTM, ovitex, synthetic mesh, resorbable mesh, and outcomes. We focused on published prospective, retrospective, cross-sectional, and case–control studies. Keywords used for the Medical Subject Heading [MeSH] search included: “Reinforced tissue matrix”[MeSH] OR “RTM”[MeSH] OR “1s permanent reinforced tissue matrix”[MeSH] OR “2s permanent reinforced tissue matrix”[MeSH] OR “ovitex”[MeSH] OR “Ovine Polymer-Reinforced Bioscaffold”[MeSH] OR “reinforced bioscaffold”[MeSH] OR “ovine derived extracellular matrix”[MeSH] OR “sheep derived extracellular matrix”[MeSH] OR “monofilament polypropylene”[MeSH] OR “Hernia” [MeSH] OR “hernias” [MeSH] OR “ventral hernia” [MeSH] OR “hiatal hernia” [MeSH]) OR “incisional hernia” [MeSH] OR “inguinal hernia” [MeSH] OR “indirect inguinal hernia” [MeSH] OR “direct inguinal hernia” [MeSH] OR “Hernia” [MeSH]. Preprint articles and abstracts accepted for publication were also considered because there is currently a lack of evidence.

### Eligibility criteria and study selection

The following criteria were used to determine which studies would be included in this review: (a) adult patients over 18 years of age; (b) hernia patients; (c) studies reporting outcomes; (d) publication of the report in English. Exclusion criteria were as follows: (a) duplicate reports (including the same patient information); (b) insufficient data; (c) animal studies; (d) reviews and reports. Two authors, SBA and KS, independently evaluated full-text articles and filtered further according to the inclusion criteria. In case of disagreements, discussions were held with the senior authors ARA and OMA until a consensus was reached.

### Data extraction

Three authors extracted variables from the information provided (author's first name, study design, publication year, sample size, country, age, sex distribution, etc.) and according to the main stratification variables (author, country, data source, mean age, age range, study period, baseline population group, total sample, and others).

### Data analysis

Statistical analysis was performed using STATA software version 17 and used the RR or with a 95% CI to estimate the risk of primary abdominal wall hernia, recurrent hernia, and open surgery between the OviTex and comparison groups. The exact binomial distribution approach was used to determine the 95% CI. The metaprop function was used to perform meta-analyses of proportions in Stata. Cochran's Q test was used to determine whether there was heterogeneity in effect sizes. A significant Q value indicates that there is heterogeneity rather than homogeneity. The I^2^ statistic was used to assess what percentage of the overall variation was attributable to study heterogeneity.^14^ Funnel plots with the Egger regression test were used to assess publication bias.^15^ We used forest plots to graphically represent the effect estimates of the included research. Statistical significance was defined as *p* < 0.05 at both ends.

### Methodology quality

We conducted quality assessments for all the included studies using the Methodological Index for Non-Randomized Studies (MINORS).^16^ The MINORS score consists of individual item scores, ranging from zero to two for each item. For comparative studies, the maximum achievable score is 24, while for non-comparative studies, it is 16 (Table 1).

### Registration

The protocol for this systematic review was registered in the PROSPERO (International Prospective Register of Systematic Reviews) with the unique number CRD42023456009.

## Results

### Study selection

**Figure 1.**
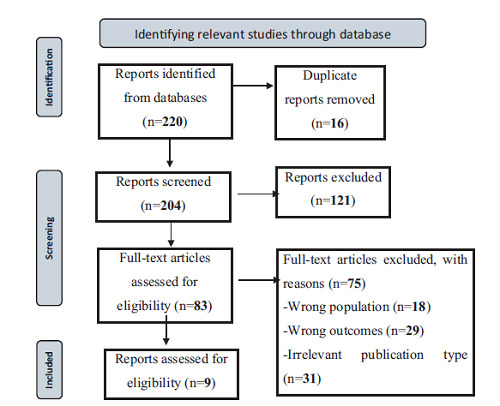
PRISMA diagram of the literature search.

A total of 220 studies were found in the literature review. Of these, 204 were excluded due to failure to meet the inclusion criteria (wrong exposure and wrong outcome). The remaining nine were incorporated into our research study (Figure 1).

### Characteristics included in the studies

A total of 523 patients from two prospective studies and six retrospective studies were included in this study. The studies were published from 2018 to 2022. We compared OviTex and other hernia treatment groups in three studies.^17–19^ (Table 1). The outcome measures of all three studies were body mass index (BMI), primary, and open surgery.

### Quality assessment

All studies included clear aims and outcomes, with either prospective or retrospective design. All studies reported a sample size calculation, and the statistical analysis was most commonly a prosthesis survival statistic (Table 1).

Two reviewers, ARA, and SBA, conducted the quality assessment of the included studies independently, and any discrepancies were resolved by mutual agreement. The MINORS scores were used prospectively and retrospectively to assess the quality of the studies. Each item was assessed and assigned a score of 0 for “not reported”, 1 for “reported but inadequate”, or 2 for “reported and adequate”. The total score was reported for each study. The MINORS score was 16 points for non-comparative studies and 24 points for comparative studies. Consequently, no studies were excluded based on methodological quality.

### Statistical analysis results

#### BMI

**Figure 2.**
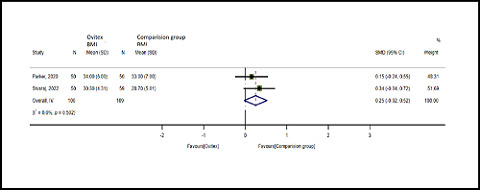
Forest plot of BMI comparison for hernia treatment.

Patients treated with OviTex had a lower BMI compared to the comparison group. One hundred patients from the OviTex group and 109 cases from the comparison group were included in the meta-analysis of reported BMI. No heterogeneity between the studies was detected by a heterogeneity test (*Q* = 0.45; *I* ^2^ = 0.0%; *p* = 0.07), and a random-effects model was used to combine the statistical data. The outcomes showed that BMI was favorable in the comparison group (Figure 2) but were not statistically significant (mean difference = 25; 95% CI: -0.02, 0.52; *p* = 0.073).

#### Primary abdominal wall hernia

**Figure 3.**
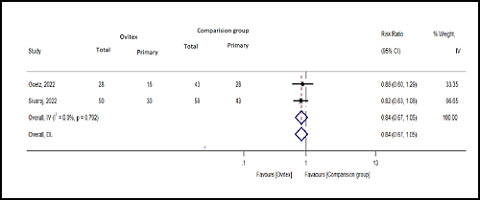
Forest plot of the primary abdominal wall hernia in the OviTex and comparison groups.

Primary abdominal wall hernia between the OviTex and comparison groups (synthetic mesh) was examined in two studies.^20–30^ The pooled risk was determined using the fixed and random-effects model. There was no heterogeneity among these studies (*I* ^2^ = 0.0%; *Q* = 0.07; *p* = 0.79). The data showed that the number of primary abdominal wall hernias was lower in the OviTex group than in the comparison group (pooled RR = 0.84; 95% CI: 0.67–1.05; Z = -1.514; *p* = 0.13) (Figure 3).

#### Recurrent hernia

**Figure 4.**
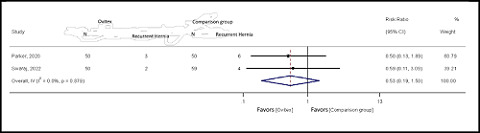
Forest plot of recurrent hernia in the OviTex and comparison groups.

Recurrent hernia between the OviTex and comparison groups was examined in two studies.^31,32^ The pooled risk was determined using the fixed-effects model. There was no heterogeneity among these studies (*I* ^2^ = 0.0%; *Q* = 0.20; *p* = 0.65). The data showed that the risk of recurrent hernia was higher in the OviTex group than in the comparison group (pooled RR = 1.36; 95% CI: 0.91–2.02; Z = 1.505; *p* = 0.132), which was not statistically significant (Figure 4).

#### Postoperative complications after open surgery

**Figure 5.**
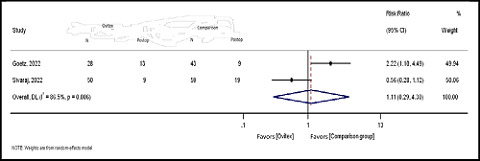
Forest plot of the postoperative risk in the OviTex and comparison groups.

Two studies examined the risk of postoperative complications after open surgery between the OviTex and comparison groups.^31,32^ There was obvious heterogeneity among these studies (*I* ^2^ = 86.5%; *Q* = 7.43; *p* = 0.006), and the pooled risk was calculated using the random-effects model. The results showed a non-significantly higher risk in the comparison group compared to the OviTex group (pooled RR = 1.11; 95% CI: 0.29–4.30; *Z* = 0.155; *p* = 0.877) (Figure 5).

#### Prevalence of primary abdominal wall hernia

**Figure 6.**
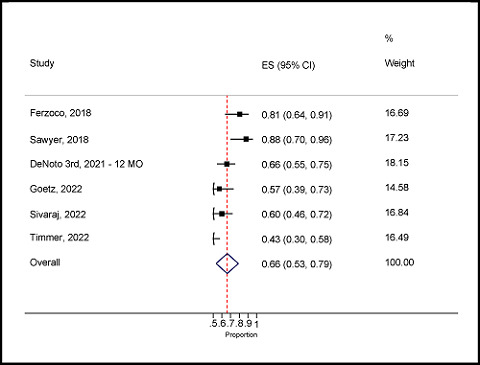
Forest plot of the proportion of primary abdominal wall hernia.

Six studies reported patients with primary abdominal wall hernia.^12,17,19,31–33^ The pooled prevalence was 66% (95% CI: 53–79%), which was statistically significant with absolute heterogeneity (*Q* = 26.7; *p* = 0.06; *I* ^2^ = 81.3%) (Figure 6). This plot shows that the prevalence rate of primary abdominal wall hernia among the included studies ranged from a minimum of 43% (95% CI: 30–58%) to a maximum of 81% (95% CI: 64–91%).

#### Prevalence of recurrent hernia

**Figure 7.**
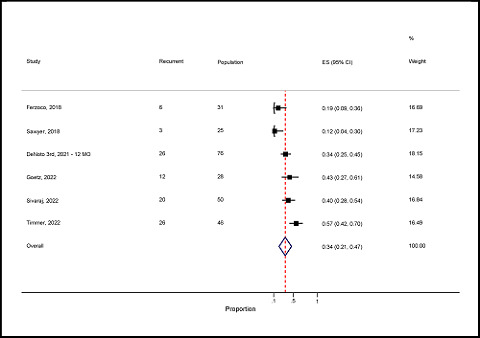
Forest plot of the proportion of recurrent hernia.

Here, we present the study-specific proportions of recurrent hernia with 95% exact confidence intervals for each study, the overall pooled estimate with 95% CIs, and the I^2^ statistic describing the percentage of total variation due to inter-study heterogeneity. The overall random-effects pooled prevalence of recurrent hernia was 34% (95% CI: 21–47%) with a high level of significant heterogeneity (*I* ^2^ = 81.3%; *p* < 0.001) (Figure 7). In addition, the minimum prevalence was 12% (95% CI: 4–30%) and the maximum prevalence was 57% (95% CI: 42–70%).

#### Prevalence of open surgery for hernia repair

**Figure 8.**
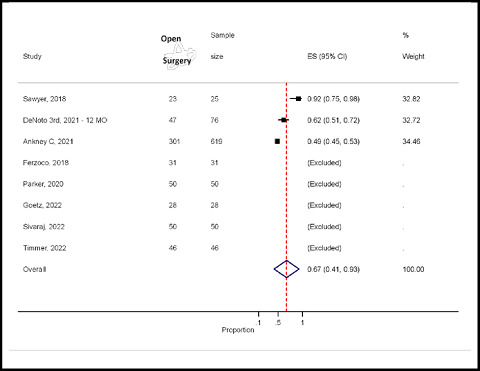
Forest plot of open surgery for hernia repair.

Seven studies reported open surgery for hernia repair.^12,17–20,31–^ ^33^ In five studies, all patients underwent open hernia surgery. Therefore, the five studies were excluded from the analysis because all the patients had open surgery.^12,17,18,31,32^ Only three studies reported open and laparoscopic surgery for hernia.^19,33,34^ The pooled prevalence of open surgery was 67% (95% CI: 41–93%), which was statistically significant (Figure 8).

#### Prevalence of laparoscopic surgery for hernia repair

**Figure 9.**
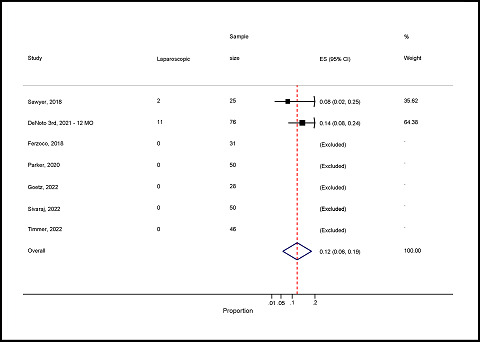
Forest plot of laparoscopic surgery for hernia repair.

The pooled prevalence of laparoscopic surgery was 12% (95% CI: 6–19%). Only two studies reported laparoscopic study of hernia.^19,33^ (Figure 9).

#### Prevalence of surgical site occurrence for hernia

**Figure 10.**
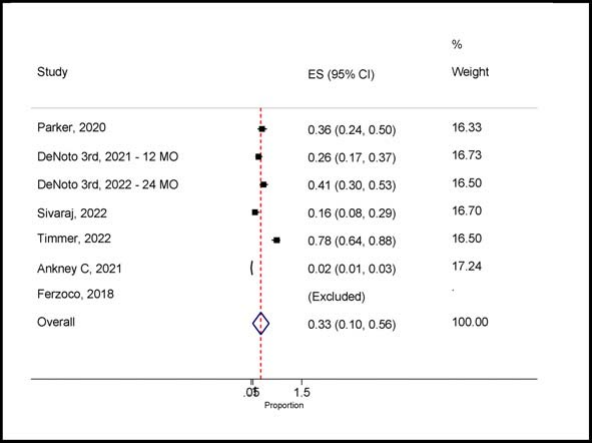
Forest plot of the surgical site occurrence for hernia.

Six studies reported the surgical site occurrence (SSO) for hernia.^2,12,18,19,32,34^ The overall random-effects pooled prevalence of SSO was 33% (95% CI: 10–56%), with a high level of significant heterogeneity (*I* ^2^ = 97.9%; *p* < 0.001) (Figure 8). In addition, the minimum prevalence was 2% (95% CI: 1–3%) and the maximum prevalence was 78% (95% CI: 64–88%) (Figure 10).

#### Prevalence of surgical site infection for hernia

**Figure 11.**
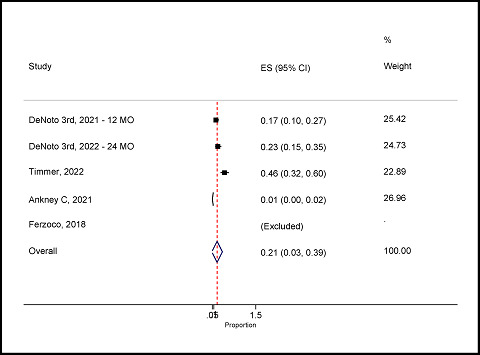
Forest plot of surgical site infection for hernia.

Four studies reported SSI for hernia.^2,12,19,34^ The overall random-effects pooled prevalence of SSI was 21% (95% CI: 3–39%) with a high level of significant heterogeneity (*I* ^2^ = 95.6%; *p* = 0.03), indicating significant variability in the reported prevalence rates across these studies. This high heterogeneity suggests differences in study populations, settings, surgical techniques, or definitions of SSIs, highlighting the importance of further research to identify and mitigate the factors contributing to SSIs in hernia patients (Figure 11).

### Publication bias

**Figure 12.**
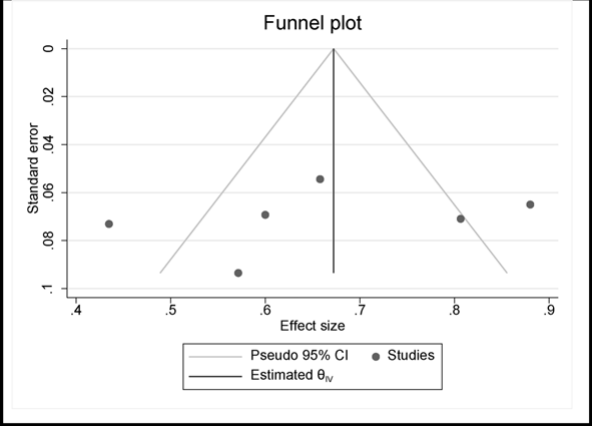
Funnel plot for detecting publication bias.

A small study impact was found in the present meta-analysis of six studies (Figure 12). The publication bias was calculated using Egger's linear regression test and was -4.38 (*p* = 0.472), which was not statistically significant. There was no concrete proof of publication bias.

## Discussion

We conducted a systematic review and meta-analysis of eight studies involving 523 patients to evaluate the effectiveness of using OviTex RTM for ventral and hiatal hernia repair. We found that the OviTex RTM shows promising outcomes in reducing recurrence rates, SSOs, SSIs, and postoperative complications.

Many types of mesh are used in hernia repair procedures, including biological and synthetic meshes.^30,35,36^ Synthetic materials such as polypropylene mesh have been widely used for hernia repair.^37^ However, concerns have been raised about capsule formation and the discomfort caused by the stiffness associated with polypropylene mesh.^37^ In addition to synthetic materials, biological materials derived from animal tissues are used in hernia mesh implants. These biological materials are specifically designed to support tissue regeneration and integration.^38^ A systematic review and meta-analysis by Figueiredo et al. (2023) compared the use of biological mesh with synthetic mesh in open ventral hernia repair.^30^ The results of the study showed no significant differences in overall complication rates between the two types of mesh. However, biological mesh was associated with a lower risk of specific complications such as seroma formation and wound infection compared to synthetic mesh. Additionally, the study found that biological mesh had a higher recurrence rate than synthetic mesh. These findings suggest that while both types of mesh are viable options for open ventral hernia repair, surgeons should consider the specific complications and recurrence rates associated with each type when deciding between the two materials.^30^ Fiber structure plays a role in determining how well the mesh material performs in hernia repair. Meshes can be categorized as monofilament or multifilament. There is no consensus on which group performs better in hernia repair.^39^ However, studies have shown that meshes with larger pores can lead to increased collagen formation and better mechanical outcomes, which, in turn, improves the organization and vascularization of the connective tissue matrix.^39^

Several studies have compared the outcomes of open and laparoscopic hernia repair, including ventral hernia repair. The studies showed that laparoscopic repair is associated with fewer perioperative complications, shorter hospital stays, and comparable recurrence rates compared to open repair.^40,41^ However, it is worth mentioning that the mesh location varied across the studies, which could potentially impact short-term complications and recurrence rates.^41^ To fully validate these findings and evaluate long-term hernia recurrence rates, further research, including randomized controlled trials with longer follow-up periods, is warranted.^41^ Apart from the type of mesh material and the surgical approach, the fixation method used in hernia repair can also influence the outcomes. A meta-analysis comparing glue and sutured mesh fixation for Lichtenstein hernia repair found that cyanoacrylate glue showed promising results with similar recurrence rates to sutured mesh fixation without any adhesive-related complications.^42^ Another meta-analysis comparing lightweight and heavyweight meshes in laparoscopic inguinal hernia procedures found that both types were associated with low recurrence rates.^43^ However, lightweight mesh was associated with less chronic pain and seroma formation.

Although the systematic review and meta-analysis showed promising results, several limitations must be considered. Variations in patient populations, surgical techniques, and follow-up duration across the included studies may have introduced heterogeneity in the results. Additionally, most of the included studies were observational, which increases the risk of selection and reporting bias. Future studies should aim to address these limitations by incorporating larger, randomized controlled trials with standardized protocols to ensure more robust comparisons.

OviTex RTM is a hybrid mesh that combines the benefits of synthetic and biological materials. It consists of an RTM composed of layers of ECM embroidered with polymer reinforcement.^19^ The biological element is derived from ovine rumen, while the polymer component is polypropylene. The OviTex material consists of six sterilized layers sewn together. This unique combination aims to provide the strength and durability of synthetic mesh while providing bioactivity and biocompatibility associated with biological mesh.^19^ The use of OviTex RTM in hernia repair has shown positive results in various studies. A study by Parker et al. compared OviTex mesh with synthetic mesh in higher-risk patients undergoing hernia repair.^18^ The findings indicated that OviTex mesh performed similarly to synthetic mesh in terms of SSOs, readmissions, and hernia recurrence. However, patients who underwent SSO with OviTex mesh were less likely to have hernia recurrence than those treated with synthetic mesh.^19^ Another study by Denoto et al. examined the clinical outcomes of OviTex 1S permanent RTM in managing ventral hernia.^19^ The study found that OviTex mesh promoted better wound healing and minimized inflammation compared to synthetic mesh. Additionally, it maintained its structural integrity and repair geometry better than many biologicals meshes. The study found that the use of OviTex 1S resulted in similar or fewer hernia recurrences and SSOs compared to traditional synthetic meshes.^19^ Timmer et al. conducted a multi-center study to evaluate the use of OviTex mesh in open abdominal wall reconstruction (AWR).^19^ Hernia recurrence rates ranged from 0 to 6%, with no mesh-related complications. A review by Sawyer et al. studied the clinical applications of reinforced tissue matrices in various hernia repair procedures. The findings of this review were consistent with the studies examined in our systematic review in terms of reinforcement, wound healing, and recurrence.

Finally, further long-term studies are needed to fully evaluate the long-term efficacy and safety of OviTex. Future research should focus on evaluating specific factors such as mesh integration, long-term recurrence rates, the impact of the material on chronic pain, and the incidence of mesh-related complications over longer follow-up periods. Such studies will provide a more comprehensive understanding of the long-term performance of OviTex and ensure its efficacy in various clinical settings.

## Limitations

To our knowledge, this is the first systematic review and meta-analysis to evaluate the use of OviTex RTM in hernia repair. However, several limitations should be acknowledged. Firstly, the included studies varied in designs, including both retrospective and prospective approaches. This variability in study design could introduce bias, as retrospective studies may have inconsistencies in data collection, while prospective studies typically follow more rigorous protocols. Exploring how these differences in design might specifically influence the results would provide a more nuanced understanding of the outcomes. Secondly, the relatively small sample sizes across the studies may limit the generalizability of our findings, as larger patient cohorts are needed to make more robust conclusions.

Additionally, some degree of heterogeneity was observed in the analyses due to differences in patient populations, surgical techniques, and follow-up duration. This variability in methodologies and populations could have an impact on the interpretation of the results and highlights the need for further standardization in future research. Furthermore, some of the included studies lacked comparison groups, making it difficult to conclusively evaluate how OviTex compares to other mesh types in terms of efficacy and safety. Despite these limitations, this systematic review provides valuable insights into the use of OviTex RTM for hernia repair. To validate these findings and fully explore the potential of OviTex, further research with larger sample sizes, standardized protocols, and longer follow-up periods is essential. Future studies should aim to address the variability in study design and the absence of comparison groups to enhance the reliability and applicability of the results.

## Conclusion

OviTex RTM has demonstrated promising outcomes in AWR and hernia repair, providing benefits such as improved wound healing, reduced inflammation, and a low incidence of hernia recurrence and surgical site complications requiring intervention. However, to fully realize its potential, more robust clinical studies are necessary. In particular, future research should focus on conducting large-scale, multi-center randomized controlled trials with standardized outcome measures to compare OviTex with other mesh materials. Additionally, longer follow-up periods are essential to assess long-term durability, recurrence rates, and patient quality of life.

Future studies should also explore specific outcomes such as the impact of OviTex on chronic pain, integration of the mesh into the tissue, and the occurrence of mesh-related complications in various patient populations, including high-risk groups. Furthermore, comparisons between OviTex and recent advances in hernia repair materials – such as lightweight meshes, self-adhering meshes, and fully resorbable meshes – will help position OviTex in the context of modern surgical options. By addressing these research gaps, the full efficacy and safety profile of OviTex can be better understood and more confidently recommended in clinical practice.

### List of abbreviations

[Table tbl2]

### Competing interests

The authors have no conflicts of interest to declare.

### Authors' contribution

**ARA**, **SBA**, **KS**, **OMA**, and **ME** performed the literature search, collected, and interpreted the data. **SBA** and **ARA** designed the work and contributed to the writing of this manuscript. **ARA** and **SBA** edited and wrote the final version of this manuscript. **ARA,** **OMA**, and **ME** reviewed the final version to be published.

### Acknowledgment

This article was funded by the Qatar National Library.

## Figures and Tables

**Table 1.** Characteristics included in the studie**s.**

**Study**	**Design**	**Comparison group**	**Type**	**Hernia treated**	**OviTex**	**Study Period**	**MINORS scores**

Ferzoco, J. 2018	Retrospective	No	NS*	Inguinal hernia	31	2016–2017	12/16

Sawyer, M, A.J., 2018	Retrospective	No	NS*	Hiatal hernia	25	2016–2017	14/16

Parker, M., et al, 2021	Retrospective	Yes	Synthetic mesh	Ventral hernia	50	2017	21/24

DeNoto, G., et al, 2021	Prospective	No	NS*	Ventral hernia	76	NS*	15/16

DeNoto, G., et al., 2022	Prospective	No	NS*	Ventral hernia	65	2017–2019	15/16

Goetz., et al., 2022	Retrospective	Yes	Resorbable meshes	Ventral hernia	28	2012–2021	20/24

Sivaraj., 2022	Retrospective	Yes	Synthetic mesh	Ventral hernia	50	2002–2021	21/24

Timmer, A., et al., 2022	Retrospective	No	NS*	Ventral hernia	46	2019–2021	13/16

Ankney C., 2021	Retrospective	No	NS*	Inguinal, ventral, Umbilical hernia	619	2015–2021	14/16


*NS: not stated.

**Table tbl2:** 

CI	Confidence Interval

MeSH	Medical Subject Heading

MINORS	Methodological Index for Non-Randomized Studies

PRISMA	Preferred Reporting Items for Systematic Reviews and Meta-Analyses

PROSPERO	International Prospective Register of Systematic Reviews

RR	Risk Ratio

RTM	Reinforced Tissue Matrix

